# The *Pseudomonas aeruginosa* CrcZ RNA interferes with Hfq-mediated riboregulation

**DOI:** 10.1371/journal.pone.0180887

**Published:** 2017-07-07

**Authors:** Elisabeth Sonnleitner, Konstantin Prindl, Udo Bläsi

**Affiliations:** Department of Microbiology, Immunobiology and Genetics, Max F. Perutz Laboratories, Center of Molecular Biology, University of Vienna, Vienna Biocenter (VBC), Vienna, Austria; Max-Planck-Institut fur terrestrische Mikrobiologie, GERMANY

## Abstract

The RNA chaperone Hfq regulates virulence and metabolism in the opportunistic pathogen *Pseudomonas aeruginosa*. During carbon catabolite repression (CCR) Hfq together with the catabolite repression control protein Crc can act as a translational repressor of catabolic genes. Upon relief of CCR, the level of the Hfq-titrating RNA CrcZ is increasing, which in turn abrogates Hfq-mediated translational repression. As the interdependence of Hfq-mediated and RNA based control mechanisms is poorly understood, we explored the possibility whether the regulatory RNA CrcZ can interfere with riboregulation. We first substantiate that the *P*. *aeruginosa* Hfq is proficient and required for riboregulation of the transcriptional activator gene *antR* by the small RNA PrrF1-2. Our studies further revealed that CrcZ can interfere with PrrF1-2/Hfq-mediated regulation of *antR*. The competition for Hfq can be rationalized by the higher affinity of Hfq for CrcZ than for *antR* mRNA.

## Introduction

Numerous studies have been performed during the last decade to decipher the function and structure of the RNA chaperone Hfq. Most studies were conducted in *E*. *coli*, and it is now well established that Hfq fulfills several functions in post-transcriptional regulation. It can stabilize small regulatory RNAs (sRNAs) and facilitate annealing between sRNAs and their target mRNAs. The latter mode of action may result either in translational repression accompanied by degradation of both RNAs or in translational activation and stabilization of the mRNA. In addition, it may stimulate polyadenylation of mRNAs, which can trigger 3´ to 5´directional decay [1]. Moreover, there is accumulating evidence that Hfq can act *per se* as a translational repressor of mRNAs [2–4].

To fulfil its role in riboregulation, the *E*. *coli* Hfq hexamer (Hfq_Ec_) has dedicated RNA binding surfaces, preferably binding uridine-rich stretches of sRNAs around the central pore of the proximal surface [5–9], and A-rich sequences, which are predominantly present around the ribosome binding sites of *E*. *coli* mRNAs [10], on the distal surface [11,12]. In agreement, amino acid (aa) exchanges in K56 located at the proximal site and Y25 located at the distal site abolished binding to poly(U) and poly(A)-tracts, respectively [7]. A third RNA binding site has been identified on the lateral rim and consists of conserved basic residues [13,14]. This basic patch binds to RNA with low sequence specificity, and appears to contribute to the annealing function of Hfq_Ec_ [14,15]. The C-terminus of Hfq_Ec_ may provide a fourth interaction site for RNA. An Hfq_Ec_ variant, comprising only the conserved core (65 N-terminal aa) was non-functional in *hfq*-autoregulation and riboregulation [16]. Moreover, biophysical experiments supported an interaction of *hfq* mRNA with the C-terminus [12,17]. In a model [8] devised to explain the role of Hfq in riboregulation it is envisioned that the mRNA binding surfaces of the Hfq-hexamer serve to transiently increase the local concentration of two RNA substrates, whereas the ability of Hfq to stochastically facilitate base-pairing is ascribed to its inherent capacity to induce conformational changes in RNAs [14,18,19].

In contrast to *E*. *coli* Hfq, the *P*. *aeruginosa* (PAO1) Hfq (Hfq_Pae_) lacks an extended C-terminus, but contains the conserved residues of the proximal and distal binding sites as well as a basic patch at the lateral rim [20]. In accordance, several reports have indicated that Hfq_Pae_ can stabilize sRNAs [21–23] as well as larger protein-binding RNAs [24]. In addition, recent *in vitro* [15] and *in vivo* assays [21,22] indicated that Hfq_Pae_ is proficient in canonical riboregulation, *i*.*e*. in sRNA-mRNA annealing.

In addition to riboregulation, our recent studies provided evidence that Hfq_Pae_ acts as the principle post-transcriptional regulator of carbon catabolite repression (CCR) in *P*. *aeruginosa* by direct binding to target mRNAs [3]. CCR ensures that the utilization of less preferred carbon sources is impeded until the preferred one is consumed [25]. During growth on succinate (CCR) Hfq was shown to be required for translational silencing of several PAO1 catabolic genes, which was attributed to an interaction of the distal face of Hfq_Pae_ with A-rich sequences within or adjacent to ribosome binding sites (RBS) [3]. Upon relief of CCR, *e*.*g*. after exhaustion of succinate and resumed growth on mannitol, the levels of the Hfq-binding RNA CrcZ increase, leading to sequestration of Hfq; this in turn abrogates Hfq-mediated translational repression of the respective catabolic mRNAs [26]. Thus, analogously to the CsrA/B/C system in *E*. *coli* and the RsmA/Y/Z system in *Pseudomonas* spp. [27], CrcZ acts as a RNA sponge for Hfq, and thus can cross-regulate catabolic genes [3].

In Enterobacteriaceae several examples are known, where regulatory sRNAs are titrated by competing endogenous RNAs. Base-pairing of sRNAs with mRNA, sRNA or even tRNA fragments other than their primary targets can interfere with regulation of the latter or reduce transcriptional noise resulting from production of sRNAs in unstressed cells [28]. As mentioned above, one alternative crosstalk is the competition of dedicated protein binding RNAs for regulatory proteins. Hierarchical binding of mRNAs can likewise exert competition for CsrA, which is exemplified by the control of fimbrial gene expression in *S*. *enterica* [29]. In *E*. *coli*, ectopic overexpression of a number of sRNAs was shown to compete with endogenous sRNAs for binding to Hfq_Ec_, which interfered with sRNA-mediated post-transcriptional regulation [30]. Similarly, over-expression of mRNA targets in the absence of the cognate regulatory sRNA was shown to diminish sRNA-mediated regulation of other target mRNAs [31]. These studies taken together with the observation that overproduction of Hfq_Ec_ could compensate for some of the regulatory defects caused by over-production of sRNAs [31] indicated that Hfq_Ec_ can be limiting for sRNA function.

As the interdependence of Hfq-mediated and RNA based control mechanisms is poorly understood in *P*. *aeruginosa*, we explored here the possibility whether the regulatory RNA CrcZ can interfere with riboregulation mediated by the sRNAs PrrF1-2. The PAO1 PrrF1-2 sRNAs are encoded in tandem, share 95% sequence identity, and are functional orthologues of the *E*. *coli* sRNA RyhB [32]. They are transcriptionally controlled by Fur, induced upon iron depletion, and implicated in post-transcriptional regulation of genes encoding functions involved in iron metabolism [32]. In addition, Oglesby *et al*. [33] showed that under iron limiting conditions the sRNAs PrrF1-2 reduce the levels of *antR* mRNA, encoding a transcriptional activator of the *antABC* operon, which is required for anthranilate degradation. It was hypothesized that this involves riboregulation by PrrF1-2 of *antR* mRNA [33].

In this study we first substantiated that Hfq_Pae_ is proficient in and required together with the PrrF1-2 sRNAs for riboregulation of *antR* mRNA. Next, we addressed the question whether the regulatory PAO1 RNA CrcZ can interfere with riboregulation of *antR* by PrrF1-2. To this end, we show that the increased synthesis of CrcZ is paralleled by de-repression of PrrF1-2-mediated regulation of *antR*. In contrast, ectopic overexpression of both *crcZ* and *hfq* resulted again in repression of *antR*, suggesting that Hfq can be limiting for PrrF1-2-mediated regulation of *antR*. We further provide evidence that the competition for Hfq can be rationalized by the higher affinity of Hfq for CrcZ than for *antR* mRNA.

## Materials and methods

### Bacterial strains, plasmids and growth conditions

The strains and plasmids used in this study are listed in S1 Table. Unless indicated otherwise, the cultures were grown at 37°C in BSM minimal medium [26] supplemented with 40 mM succinate. If required, *E*. *coli* was grown in the presence of 100 μg ml^-1^ ampicillin, 25 μg ml^-1^ tetracycline or 15 μg ml^-1^ gentamicin. PAO1 was grown in the presence of 250 μg ml^-1^ carbenicillin, 100 μg ml^-1^ tetracycline or 50 μg ml^-1^ gentamicin, respectively. The construction of plasmids used in this study is described in S1 Text.

### β-galactosidase assays

The β-galactosidase activities were determined as described by Miller [34]. The cells were permeabilized with 5% toluene. The β-galactosidase activities in either experiment are derived from three independent experiments. The translational efficiency was determined by normalization of the β-galactosidase activities derived from the translational *lacZ* gene fusions to the values derived from the transcriptional *lacZ* gene fusions. The error bars in the different Figures represent standard deviations.

### Northern-blot analyses

Total RNA was purified using hot phenol [35]. The steady state levels of PrrF sRNA, CrcZ and 5S rRNA (loading control) were determined by Northern-blotting using 4 μg of total RNA. The RNA samples were denatured for 5 min at 65°C in loading buffer containing 50% formamide, separated on a 8% polyacrylamide/8 M urea gel, and then transferred to a nylon membrane by electro-blotting. The RNA was cross-linked to the membrane by exposure to UV light. The membranes were hybridized with gene-specific ^32^P-end-labelled oligonucleotides (PrrF: U32 (5’-GTG ATT AGC CTG ATG AGG AG-3); CrcZ: K3 (5’-GCT GGA GTC GTT ACG TGT TG-3’); 5S rRNA: I26 (5’-CCC CAC ACT ACC ATC GGC GAT GCG TCG-3’). The hybridization signals were visualized using a PhosphorImager (Molecular Dynamics).

### Protein purification

The Hfq_Pae_, Hfq_PaeY25DFlag_ and Hfq_PaeK56A_ proteins were produced in the *hfq* deficient *E*. *coli* strain AM111F′ harboring the plasmids pHfq_Pae_, pHfq_PaeY25DFlag_ and pHfq_PaeK56A_, respectively. The protein purifications were performed as described by Beich-Frandsen *et al*. [17]. Hfq_Y25DFlag_ was used as it showed increased stability when compared to the untagged version.

### Western-blot analyses

Equal amounts of total proteins were separated on 12% SDS-polyacrylamide gels and electro-blotted to a nitrocellulose membrane. The blots were blocked with 5% dry milk in TBS buffer, and then probed with rabbit anti-Hfq (Pineda), rabbit anti-Flag (Roth) or rabbit anti-S1 (control) antibodies. The antibody-antigen complexes were visualized with alkaline-phosphatase conjugated secondary antibodies (Sigma) using the chromogenic substrates nitro blue tetrazolium chloride (NBT) and 5-Bromo-4-chloro-3-indolyl phosphate (BCIP).

### *In vitro* transcription

For *in vitro* transcription of *antR*_(-95–+67)_ (162 nt), PrrF2 (107 nt), and CrcZ (426 nt) RNAs the AmpliScribe T7-Flash Transcription Kit (Epicentre Biotechnologies) was used according to the manufacturer´s instructions. First, PCR fragments were generated with the primer pairs T94 (5’-TGC TCT AGA **CGT AAT ACG ACT CAC TAT AG**G GAG CCG GCC TTG CG-3’)/U94 (5’-CGG CGG CCA GGT CCA GG-3’) (*antR*), W77 (5’- TTT TCT AGA **CGT AAT ACG ACT CAC TAT AGG** ACT GGT CGC GAG GCC-3’)/X77 (5’-CAA AAA AAG ACC CGG CAA AG-3‘) (PrrF2), and E6 (5’-TCT AGA **CGT AAT ACG ACT CACT ATA GG**C ACA ACA ACA ATA ACA AGC -3’)/C6 (5’-ATG CGG ATC CGA AAT GGT GTA AGG CGA AGG -3’) (CrcZ). The forward primers contained T7 promoter sequences, which are shown in bold.

### Electro mobility shift assays

**Fig 1:**
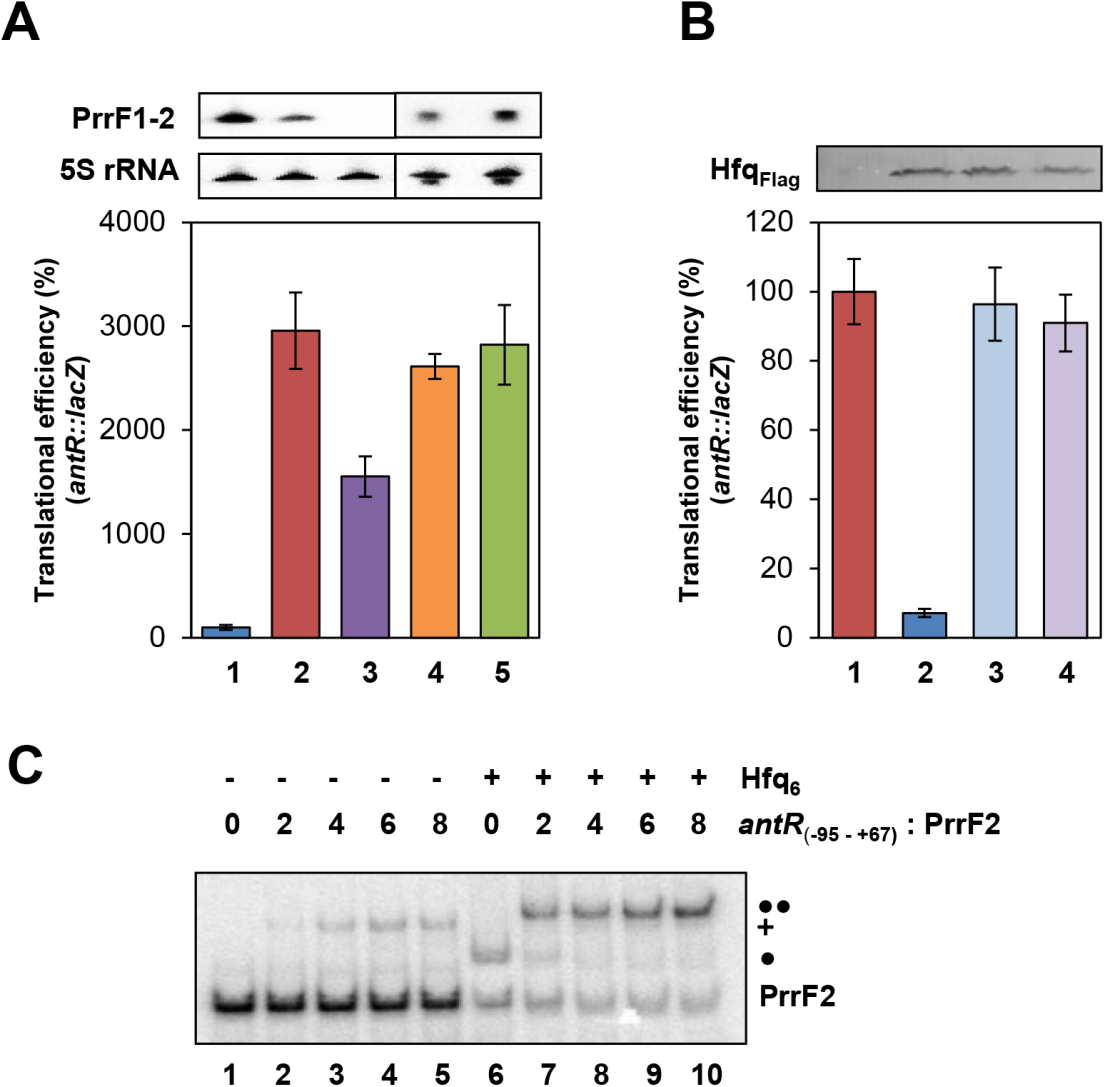
Hfq_Pae_ performs canonical riboregulation. (A) Repression of *antR* by Hfq and PrrF1-2 sRNA. The strains were grown in BSM medium supplemented with 40 mM succinate and 0.2 mM anthranilate (to induce *antR* transcription) to an OD_600_ of 2.0. The cells were then harvested and the β-galactosidase activities were determined. The bars depict the translational efficiency conferred by the translational *antR*::*lacZ* fusion, encoded by plasmid pTLantR, normalized to the β-galactosidase values obtained with the transcriptional *antR-lacZ* fusion, encoded by plasmid pTCantR2, in strains PAO1 (lane 1), PAO1Δ*hfq* (lane 2), PAO1Δ*prrF1-2* (lane 3), PAO1Δ*hfq* (pME4510) (lane 4) and PAO1Δ*hfq* (pP_tac_PrrF2) (lane 5), respectively. The translational efficiency conferred by the translational *antR*::*lacZ* fusion in strain PAO1(pTLantR) (lane 1) was set to 100%. The error bars represent standard deviations from three independent experiments. The PrrF levels (top panel) were determined by Northern-blot analysis. 5S rRNA served as a loading control. (B) The distal and the proximal binding surfaces of Hfq are required for *antR* repression. The cultures were grown in BSM medium supplemented with 40 mM succinate and 0.2 mM anthranilate to an OD_600_ of 2.0. The translational efficiency conferred by the translational *antR*::*lacZ* fusion was determined in strain PAO1Δ*hfq* (pTLantR) harboring the control plasmid pME4510 (lane 1), plasmid pME4510hfq_Flag_ (lane 2), plasmid pME4510hfq_Y25DFlag_ (lane 3) and pME4510hfq_K56AFlag_ (lane 4), respectively. The translational efficiency conferred by the translational *antR*::*lacZ* fusion in strain PAO1Δ*hfq*(pME4510,pTLantR) was set to 100%. The error bars represent standard deviations from three independent experiments. The protein levels of Hfq_Flag_, Hfq_Y25DFlag_ and Hfq_K56AFlag_ (top panel) were determined by western-blot analysis using anti-Flag antibodies. (C) Hfq accelerates PrrF**-***antR* duplex formation. 10 nM radioactively labeled PrrF2 RNA was incubated alone (lane 1) or with increasing amounts of *antR*_(-95–+67)_ (2, 4, 6 and 8-fold molar excess) in the absence (lanes 2–5) or presence (lanes 6–10) of Hfq (the molar ratio of PrrF2 RNA to Hfq-hexamer was 1:8), and the resulting complexes were analyzed on a 4% native polyacrylamide gel. Single and double circles denote the PrrF2**·**Hfq and PrrF2**·**Hfq**·***antR*_(-95–+67)_ complexes, respectively. The plus symbol denotes the PrrF2**·***antR*_(-95–+67)_ complex.

The *antR*_(-95–+67)_ and PrrF2 RNAs were de-phosphorylated with FastAP thermo sensitive alkaline phosphatase (Thermo Scientific), and subsequently 5´-end labeled using [γ-^32^P]-ATP (Hartmann Analytic) and polynucleotide kinase (Thermo Scientific). The labelled RNAs were gel-purified following standard procedures, eluted and kept in diethylpyrocarbonate- (DEPC) treated water. Labeled RNA (0.1 pmol) dissolved in DEPC water was incubated with increasing amounts of Hfq_Pae_, Hfq_PaeY25DFlag_ and Hfq_PaeK56A_ proteins (S3 Fig) or unlabeled RNA and Hfq (Figs 1C and S5) in 10 mM Tris-HCl (pH 8.0), 10 mM MgCl_2_, 60 mM NaCl, 10 mM NaH_2_PO_4_, 10 mM DTT, and 25 ng tRNA in a total volume of 10 μl. The reaction mixtures were incubated at 37°C for 30 min to allow protein–RNA complex formation. The samples were mixed with 4 μl loading dye (25% glycerol, 0.2 mg/l xylencyanol and bromphenol blue) immediately before loading and separated on 4% polyacrylamide gels using Tris-borate buffer. The radioactively labeled bands were visualized with a PhosphorImager (Molecular Dynamics) and quantified with ImageQuant software 5.2.

### Microscale thermophoresis

Binding of CrcZ and *antR*_(-95–+67)_ to Hfq, respectively, was determined by microscale thermophoresis (MST) [36] using the Monolith NT.115 Blue/Red apparatus (Nanotemper Technologies) at the Protein Technologies Facility (ProTech, VBCF, Vienna, Austria). Labelling of 20 μM of Hfq_Pae_ protein with NT-642 dye was performed using the Monolith™ Antibody Labelling Kit RED-NHS (Amine Reactive; Nanotemper Technologies) according to the manufacturer's instructions. For the measurements, the concentration of the NT-642-labelled Hfq protein was kept constant (30 nM for CrcZ binding and 20 nM for *antR*_(-95–+67)_ binding), whereas the concentrations of non-labelled *in vitro* transcribed CrcZ and *antR*_(-95–+67)_ RNA varied from 0.03 to 250 nM (CrcZ) and from 0.25 to 500 nM (*antR*_(-95–+67)_). The binding reactions were carried out in ES-buffer (10 mM Tris pH 8.0, 40 mM NaCl, 10 mM KCl, 1 mM MgCl_2_) supplemented with 0.05% Tween. The reactants were initially incubated at room temperature for 5 min to enable RNA binding by Hfq. The samples were then loaded onto NT.115 MST premium coated capillaries (Nanotemper Technologies). The data for microscale thermophoresis analysis were recorded at 25°C using the red LED (excitation: 625 nm, emission: 680 nm); MST and LED Power was set for CrcZ/Hfq at 20% and 80%, respectively, and for *antR*_(-95–+67)_/Hfq at 60% and 90%, respectively. Data analyses were performed with NTAnalysis software (Nanotemper Technologies).

## Results and discussion

### Hfq_Pae_ functions in riboregulation: It stabilizes PrrF1-2 sRNAs and stimulates base-pairing between the sRNA PrrF2 and *antR* mRNA

Previous studies revealed that PrrF1-2 binds to Hfq [37] of *P*. *aeruginosa* and that the *antR* levels were increased in the absence of Hfq [24] and of PrrF1-2 [33]_,_ respectively. Taken with the *in silico* prediction of complementarity between PrrF1-2 RNAs and the translation initiation site of *antR* mRNA [33], these observations prompted us to adopt the PrrF1-2/*antR* entity to substantiate that Hfq_Pae_ executes canonical riboregulation, i.e. facilitating base-pairing between a sRNA and its mRNA target.

We first used transcriptional and translational reporter genes to test the influence of Hfq_Pae_ on PrrF1-2 mediated repression of *antR*. The strains were grown in BSM medium supplemented with 40 mM succinate and 2 μM FeSO_4_, which resulted in PrrF1-2 expression (Fig 1A, upper panel, atop lane 1). When compared with the wild-type strain no significant difference in the β-galactosidase activity conferred by the transcriptional *antR-lacZ* fusion gene was observed in the PAO1Δ*hfq* strain (S1 Fig). At variance, despite the presence of PrrF1-2, the absence of Hfq_Pae_ resulted in de-repression of *antR*::*lacZ* translational efficiency (Fig 1A, lane 2). Interestingly, the absence of PrrF1-2 but presence of Hfq in strain PAO1Δ*prrF1-2* did not abolish repression to the same extend as seen in the absence of Hfq alone (Fi.g 1A, lane 3). Given that Hfq_Pae_ has been shown to act as a translational repressor on several mRNAs [3], we speculate that Hfq_Pae_ *per se* represses *antR*::*lacZ* translation, albeit less efficiently than in combination with PrrF1-2. Although Hfq_Pae_ is also required for stabilization of PrrF1-2 (S2 Fig), additional ectopic expression of *prrF2* did not result in *antR*::*lacZ* repression in the absence of Hfq_Pae_ (Fig 1A, lane 5), indicating that the RNA chaperone is required for PrrF1-2/*antR* annealing.

As anticipated for canonical riboregulation [1], we next asked whether the proximal and distal interaction sites of Hfq_Pae_ are required for binding of PrrF2 sRNA and *antR* mRNA, respectively. As shown in S3A Fig, the Hfq_PaeY25D_ variant, which is deficient in distal site binding of A-rich sequences [7], did not bind to the *antR*_(-95–+67)_ mRNA fragment encompassing the translational initiation region (TIR). Likewise, the Hfq_PaeK56A_ mutant protein, which is defective in binding to U-rich sequences of sRNAs [7] was deficient in binding to PrrF2 RNA (S3B Fig). These EMSA assays are in agreement with the observation that ectopic expression of the *hfq*_PaeY25D_ and *hfq*_PaeK56A_ mutant alleles did not lead to repression of *antR*::*lacZ* translation (Fig 1B, lanes 3 and 4).

Our studies (S4 Fig) and studies performed by Zheng *et al*. [15] showed that Hfq_Pae_ stimulates annealing of complementary ribo-oligonucleotides, albeit to a reduced extend when compared with Hfq_Ec._ The latter finding has been ascribed to the increased arginine content in the basic patch on the rim of Hfq_Ec_ when compared with Hfq_Pae_ [15]. To test whether Hfq stimulates annealing of PrrF2/*antR*_(-95–+67)_ an EMSA assay was employed. Radioactively labelled PrrF2 RNA was incubated with increasing amounts of *antR*_(-95–+67)_ mRNA in the absence (Fig 1C, lanes 2–5) or presence (Fig 1C, lanes 7–10) of Hfq_Pae_, and the resulting complexes were analyzed on native polyacrylamide gels. As shown in Fig 1C, lanes 7–10, PrrF2**·***antR*_(-95–+67)_ complex formation was strongly accelerated by Hfq_Pae_. To test whether the complex indicated with 2 circles (Fig 1C) indeed contained Hfq, selected samples, which are shown in lanes 1, 5, 6, and 7 of Fig 1C, were resolved on a separate gel and a western-blot was performed. As revealed by immuno-detection, Hfq was part of the PrrF2**·***antR*_(-95–+67)_ complex (S5A Fig).

*Vice versa*, labelling of *antR*_(-95–+67)_ mRNA confirmed that base pairing with PrrF2 is inefficient in the absence of Hfq_Pae_ (S5B Fig, lanes 2–5 and lanes 7–10). In summary, these studies substantiated that Hfq_Pae_ -like Hfq_Ec_- can perform canonical riboregulation, and thus established the PrrF1-2/*antR* entity as a suitable model system to study the interdependence of RNA-mediated regulation in PAO1.

### The regulatory RNA CrcZ competes for Hfq and interferes with *antR* translation

**Fig 2:**
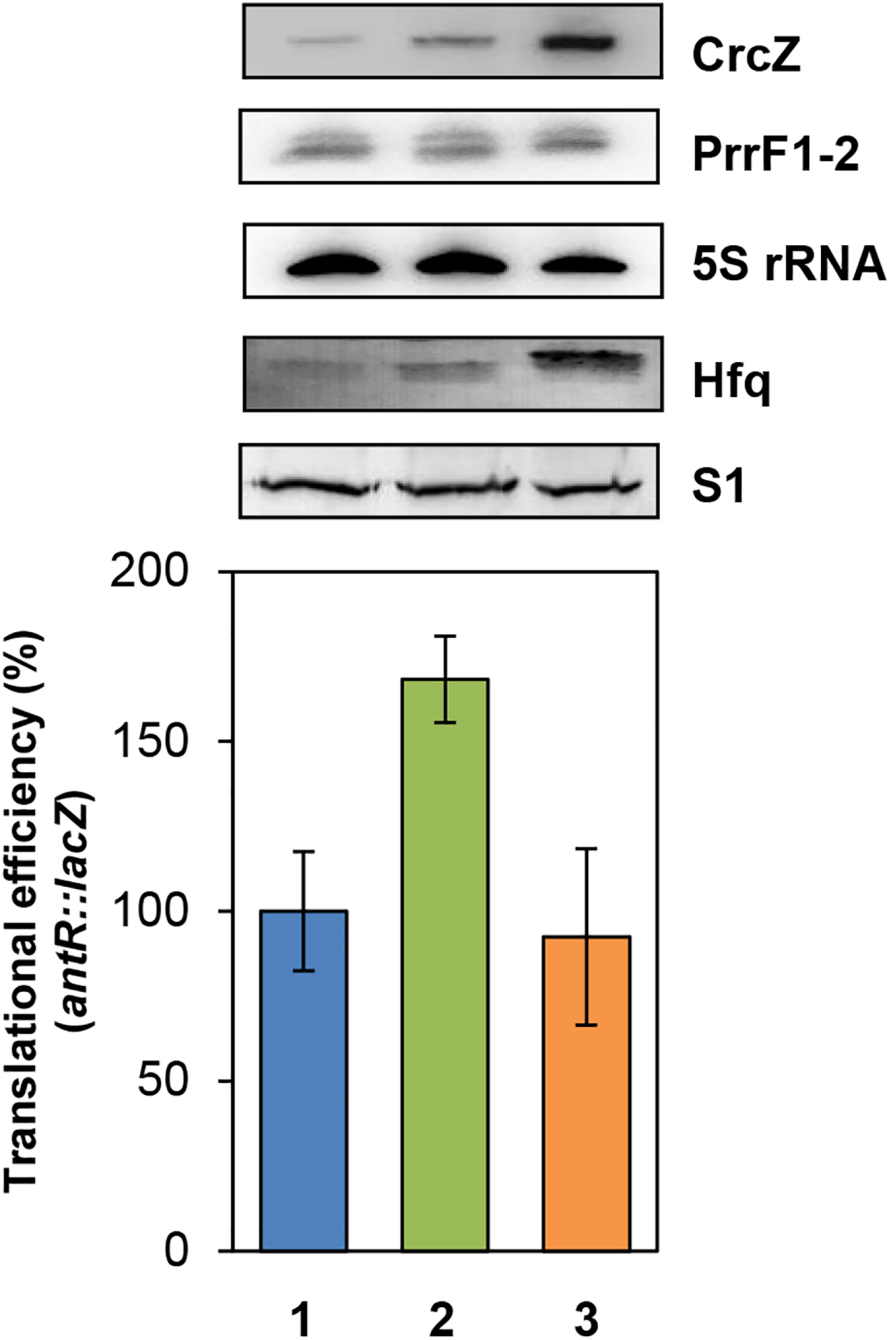
Ectopic expression of *crcZ* results in de-repression of *antR*::*lacZ* translation. The strains PAO1(pMMB67HE,pME4510,pTLantR), PAO1(pMMBcrcZ,pME4510,pTLantR), PAO1(pMMBcrcZ,pME4510hfq_Flag_,pTLantR), PAO1(pMMB67HE,pME4510,pTCantR2), PAO1(pMMBcrcZ,pME4510,pTCantR2) and PAO1(pMMBcrcZ, pME4510hfq_Flag_,pTCantR2) were grown in BSM medium supplemented with 40 mM succinate and 0.2 mM anthranilate to an OD_600_ of 2.0. The cells were then harvested and the β-galactosidase activities conferred by the transcriptional *antR-lacZ* fusion gene and the translational *antR*::*lacZ* fusion gene, encoded by plasmids pTCantR2 and pTLantR, respectively, were determined in lysates of the respective strains. The β-galactosidase values obtained with the translational *antR*::*lacZ* fusion genes were normalized to that obtained with the transcriptional *antR-lacZ* fusion genes. The bars depict the translational efficiency of the *antR*::*lacZ* gene encoded by plasmid pTLantR in strain PAO1(pMMB67HE,pME4510, pTLantR) (lane1, blue bar), PAO1(pMMBcrcZ,pME4510,pTLantR) (lane 2, green bar) and PAO1(pMMBcrcZ,pME4510hfq_Flag_,pTLantR) (lane 3, orange bar), respectively. The translational efficiency conferred by the translational *antR*::*lacZ* fusion in strain PAO1(pMMB67HE,pME4510,pTLantR) was set to 100%. The error bars represent standard deviations from three independent experiments. The CrcZ and PrrF1-2 levels (top panels) were determined by Northern-blot analysis. 5S rRNA served as a loading control. The Hfq and S1 (loading control) levels were determined by western-blotting.

As both *antR*_(-95–+67)_ (S3A Fig) and CrcZ [3] bind to the distal face of Hfq, we next tested whether ectopic overexpression of *crcZ* interferes with PrrF1-2-mediated regulation of *antR*. As shown in Fig 2 (lane 2), elevated levels of CrcZ increased the translational efficiency of the *antR*::*lacZ* gene approximately 1.7-fold, indicating that CrcZ can interfere with PrrF1-2/*antR* riboregulation. In agreement, overexpression of *crcZ* and *hfq* resulted in a similar repression of *antR*::*lacZ* as under non-induced conditions (Fig 2, lanes 1 and 3). This suggested that Hfq was limiting for PrrF1-2 function in the experiment shown in Fig 2 (lane 2).

**Fig 3:**
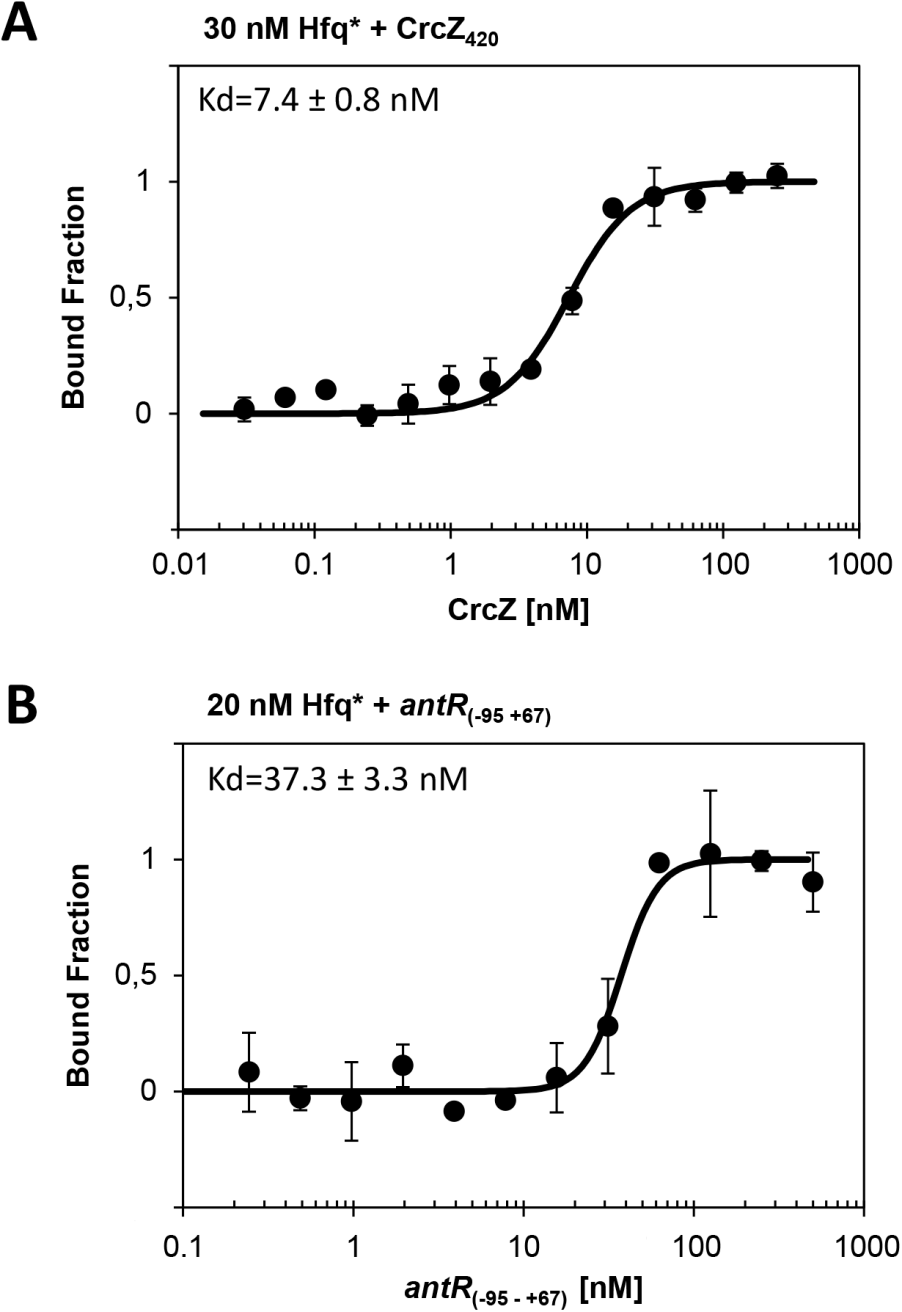
**K**_**d**_ **of Hfq for CrcZ (A) and *antR***_**(-95–+67)**_ **(B) RNAs revealed by microscale thermophoresis.** Increasing amounts of non-labelled *in vitro* transcribed CrcZ or *antR*_(-95–+67)_ RNAs were added to 30 nM (A) and 20 nM (B) fluorescently labelled Hfq protein, respectively. The dissociation constants (K_d_) of CrcZ and *antR*_(-95–+67)_ were expressed as mean EC50 ± EC50 confidence interval of 2 independent experiments.

To rationalize the competition for Hfq_Pae_ by CrcZ, we next determined the affinity of Hfq_Pae_ for CrcZ and *antR*_(-95–+67)_ using microscale thermophoresis. The K_d_ was determined with ~ 7.4 nM and ~ 37.3 nM for CrcZ and *antR*_(-95–+67)_ RNA, respectively (Fig 3). The low K_d_ of Hfq_Pae_ for CrcZ can be reconciled with six A-rich stretches in CrcZ to which Hfq_Pae_ can bind with its distal binding site(s) [3]. Hence, it seems reasonable to assume that CrcZ can efficiently compete for Hfq_Pae_ as long as the affinity of the distal binding surface of Hfq_Pae_ for any given (m)RNA is lower than for CrcZ. This result is in agreement with the recent finding that CrcZ was shown to compete for the distal site of Hfq_Pae_ with *amiE* mRNA to which Hfq binds with a K_d_ of ~ 67.0 nM [3].

**Fig 4:**
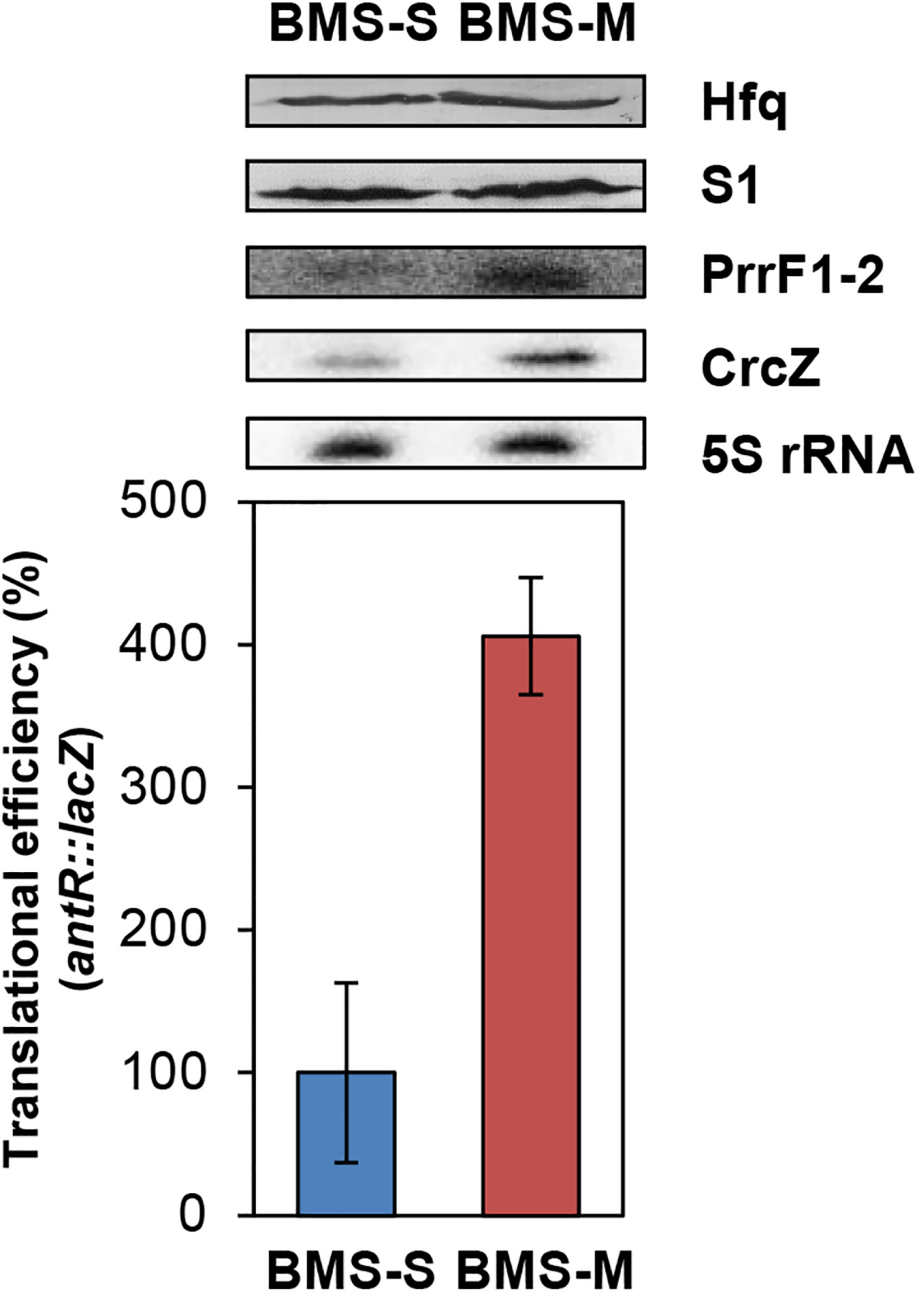
Increasing CrcZ levels lead to de-repression of *antR*::*lacZ* translation. PAO1(pTLantR) and PAO1(pTCantR2) were grown to an OD_600_ of 0.5 in BSM medium supplemented with 5 mM succinate, 0.2 mM anthranilate (blue bar) and 5 mM mannitol, 0.2 mM anthranilate (red bar), respectively. Then, the cells were harvested and the β-galactosidase activities were determined. The bars depict the translational efficiency conferred by the translational *antR*::*lacZ* fusion, encoded by plasmid pTLantR, normalized to the β-galactosidase values obtained with the transcriptional *antR-lacZ* fusion, encoded by plasmid pTCantR2. The translational efficiency conferred by the translational *antR*::*lacZ* fusion in strain PAO1(pTLantR) in BSM-S was set to 100%. The error bars represent standard deviations from three independent experiments. Top: The PrrF1-2 and CrcZ levels were determined by Northern-blot analyses. 5S rRNA served as a loading control. The Hfq and S1 (loading control) levels were determined by western-blotting as described in Materials and methods.

Next, we asked whether this interference is also apparent under conditions that are closer to physiology. Given that the CrcZ levels increase in poor carbon sources, *e*.*g*. mannitol [26], the strain PAO1(pTLantR), harboring the plasmid borne *antR*::*lacZ* translational reporter gene, and the strain PAO1(pTCantR2), harboring the plasmid borne *antR*-*lacZ* transcriptional reporter gene, were grown in BSM medium containing either succinate (BSM-S) or mannitol (BSM-M). As shown in Fig 4, when compared with growth in BSM-S, the Hfq levels were unaltered and the CrcZ levels were increased approximately 2.7-fold in BSM-M. Under these conditions, the translational efficiency of the *antR*::*lacZ* gene in strain PAO1 was de-repressed during growth in BSM-M when compared with growth in BSM-S (Fig 4). It should be noted that de-repression occurred despite the PrrF1-2 levels were—for unknown reasons—increased. Therefore, we concluded that this observation is attributable to the higher levels of CrcZ observed in BSM-M, *i*.*e*. to titration of Hfq by CrcZ.

**Fig 5:**
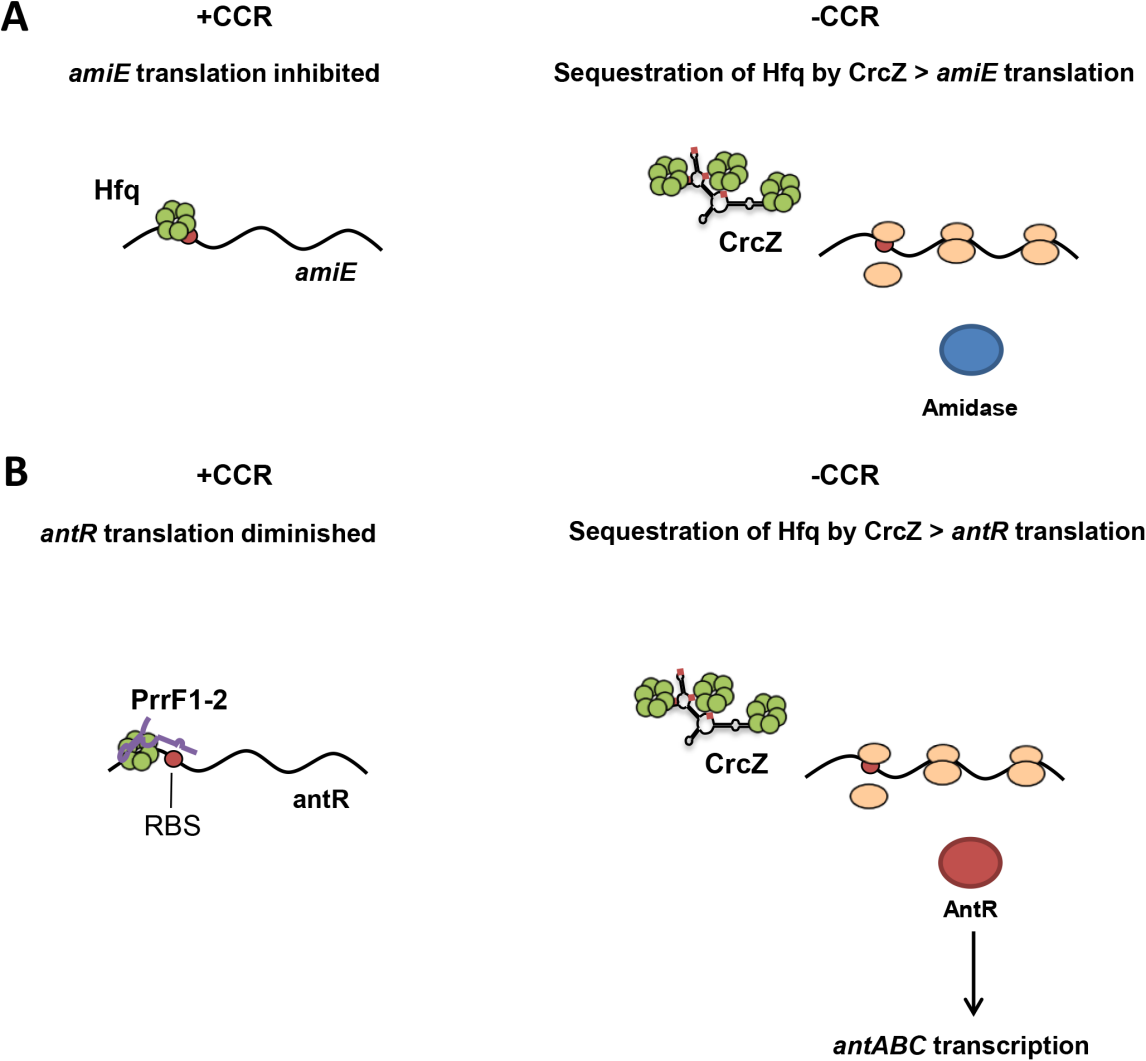
Cross-regulation by CrcZ in Hfq-mediated translational control. (A) Canonical function of CrcZ during carbon catabolite repression (CCR). Translational repression by Hfq of catabolic mRNAs (*e*.*g*. *amiE*) during growth on preferred carbon sources, *e*.*g*. succinate (+CCR; left), is relieved upon accumulation of the Hfq-titrating RNA CrcZ during growth on poor carbon sources, *e*.*g*. amides (-CCR; right) [3]. (B) CrcZ cross-regulates mRNAs subjected to Hfq-mediated riboregulation. During iron limitation and growth on preferred carbon sources *antR* translation is repressed by PrrF1-2 and Hfq (+CCR; left). Upon relief of CCR (-CCR; right), CrcZ competes for Hfq binding, which interferes with PrrF1-2/Hfq-mediated riboregulation, which in turn can activate *antR* translation (right), and consequently anthranilate degradation.

We have recently reported that competition for Hfq by CrcZ relieves translational repression of direct Hfq target mRNAs, *e*.*g*. *amiE* [3] (Fig 5A). This study extends the assigned role of Hfq and CrcZ in CCR of *P*. *aeruginosa*. Here, the regulatory circuit is more complex in that CrcZ most likely interferes with negative riboregulation, i.e. with PrrF1-2/Hfq-mediated repression of *antR* mRNA (Fig 5B). Consequently, the transcriptional regulator AntR is synthesized and can induce transcription of the *antABC* operon required for anthranilate degradation. As CrcZ can efficiently compete for Hfq, transcriptome studies are currently underway to identify further Hfq controlled pathways that are cross-regulated by CrcZ.

## Supporting information

S1 Table Strains and plasmids used in this study. (DOCX)

S1 Text Supporting materials and methods. (DOCX)

S1 Fig Hfq does not impact on *antR* transcription. The strains PAO1(pTCantR2) and PAO1Δ*hfq*(pTCantR2) were grown in BSM medium supplemented with 40 mM succinate and 0.2 mM anthranilate. When compared with the wild-type strain (blue bar) no significant difference in the β-galactosidase activity conferred by the transcriptional *antR-lacZ* fusion gene was observed in the PAO1Δ*hfq* strain (red bar).
(TIF)

S2 Fig Hfq stabilizes PrrF1-2 RNA. PAO1 and PAO1Δ*hfq* were grown in BSM medium supplemented with 40 mM succinate. At an OD_600_ of 1.5, rifampicin was added to a final concentration of 100 μg/ml and samples were withdrawn for total RNA extraction at the times indicated. (A) The levels of PrrF1-2 and 5S rRNA (loading control) were determined by Northern-blot analyses with oligonucleotides specific for either RNA as described in Materials and methods. The result from one representative experiment is shown. (B) Graphical representation of the results. The concentrations of PrrF1-2 RNA in PAO1 (blue diamonds) and PAO1Δ*hfq* (red diamonds), respectively, were normalized to that of 5S rRNA at different times after addition of rifampicin. The results are derived from two independent experiments. Error bars represent standard deviations. The half-life of PrrF1-2 RNA was determined with 12 ± 3 min in PAO1 and 5 ± 1 min in PAO1Δ*hfq*.
(TIF)

S3 Fig The distal and the proximal sites of Hfq are required for *antR* and PrrF1-2 binding. (A) EMSA with 10 nM radioactively labeled *antR*_(-95–+67)_ mRNA in the absence (lane 1) and in the presence of 1-fold (lanes 2, 5 and 8), 4-fold (lanes 3, 6 and 9) and 8-fold (lanes 4, 7 and 10) molar excess of Hfq_Pae_ (Hfq_wt_), Hfq_PaeY25DFlag_ and Hfq_PaeK56A_, respectively. (B) EMSA with 10 nM radioactively labeled PrrF2 sRNA in the absence (lane 1) and in the presence of 4-fold (lanes 2, 5 and 8), 8-fold (lanes 3, 6 and 9) and 16-fold (lanes 4, 7 and 10) molar excess of Hfq_Pae_ (Hfq_wt_), Hfq_PaeY25DFlag_ and Hfq_PaeK56A_, respectively.
(TIF)

S4 Fig Hfq_Pae_ accelerates RNA annealing. RNA annealing activities of Hfq_Pae_ and Hfq_Ec_. 5 nM single stranded, complementary 21-nt-long oligonucleotides with fluorophores at their 5’-end were annealed at 37°C in the absence (red bar) or presence of 100 nM Hfq_Ec_ (green bar) and Hfq_Pae_ (blue bar) protein, respectively. Relative fluorescence resonance energy transfer (FRET) was calculated as ratio of acceptor to donor fluorescence (F_Cy5_/F_Cy3_) as described in S1 Text. The time-resolved curves were least-square fitted with the second-order reaction equation for equimolar initial reactant concentrations: *y* = *A*[1-(*k*_*obs*_ *t*+1)^-1^]; *y* = fraction annealed, *k*_*obs*_ = observed annealing reaction constant, *A* = maximum reaction amplitude. The reaction rate *k*_*obs*_ was calculated from the average of three independent experiments.
(TIF)

S5 Fig Analysis of PrrF2·Hfq·*antR*_(-95–+67)_ complexes. (A) Left panel: The samples corresponding to those shown in Fig 1C, lanes 1, 5, 6 and 7 were resolved on a separate 4% native polyacrylamide gel. Single and double circles denote the PrrF2**·**Hfq and PrrF2**·**Hfq**·***antR*_(-95–+67)_ complexes, respectively. The plus symbol denotes the PrrF2**·***antR*_(-95–+67)_ complex. Right panel: Western-blot of the gel shown at the left probed with anti-Hfq antibodies. The presence of Hfq in the PrrF2**·**Hfq and PrrF2**·**Hfq**·***antR*_(-95–+67)_ complexes is indicated by single and double circles, respectively. (B) Hfq accelerates PrrF**-***antR* duplex formation. 10 nM radioactively labeled *antR*_(-95–+67)_ RNA was incubated alone (lane 1), with Hfq_Pae_ (lane 6) or with increasing amounts of PrrF2 (2, 4, 6 and 8-fold molar excess) in the absence (lanes 2–5) or presence (lanes 6–10) of Hfq_Pae_ (the molar ratio of Hfq_Pae_-hexamer to *antR*_(-95–+67)_ RNA to was 2:1), and the resulting complexes were analyzed on a 4% native polyacrylamide gel. Single and double circles denote the *antR*_(-95–+67)_**·**Hfq and PrrF2 Hfq**·***antR*_(-95–+67)_ complexes, respectively. The plus symbol denotes the PrrF2**·***antR*_(-95–+67)_ complex.
(TIF)
