# Supplementary material for: The Pseudomonas aeruginosa CrcZ RNA interferes with Hfq-mediated riboregulation
Source: PLoS One. 2017 Jul 7;12(7):e0180887. doi: 10.1371/journal.pone.0180887 (PMC5501646; doi:10.1371/journal.pone.0180887)
Supplement: S1 Table — (DOCX) [file pone.0180887.s001.docx]

**S1 Table.** **Strains and Plasmids used in this study**

| **Strains/plasmid** | **Genotype/relevant feature** | **Source/reference** |
| --- | --- | --- |
| ***P. aeruginosa*** |  |  |
| PAO1 |  | [42] |
| PAO1Δ*hfq* | deletion of base pairs 5548400- 5548645 [38] | This study |
| PAO1Δ*prrF1-2* | deletion of base pairs 5283933-5284353 [38] | [32] |
| PAO1Δ*crcZ* | PAO6679, deletion of base pairs 5308523-5308682 [38] | [26] |
| ***E. coli*** |  |  |
| AM111F’ | AM111 [F‘ *proAB* *lacI^q^* *lacZ*ΔM15::Tn*10*; Tc^r^] | [20] |
| XL1Blue | *recA1 endA1 gyrA96 thi-1 hsdR17*(r_K_-, m_K_+) *supE44 relA1 lac* [F' *proAB lacI^q^lacZ*ΔM15::Tn*10*(Tc^r^)] | Stratagene |
| **Plasmids** |  |  |
| pME6016 | Cloning vectors for transcriptional *lacZ* fusions (pME6010 derivative); Tc^r^ | [43] |
| pME6014 | Cloning vectors for translational *lacZ* fusions (pME6010 derivative); Tc^r^ | [43] |
| pTLantR | pME6014 derivative with a translational *lacZ* fusion of *antR* | This study |
| pTCantR2 | pME6016 derivative with a transcriptional *lacZ* fusion of *antR* | This study |
| pME4510 | Broad–host-range promoter-probe plasmid; Gm^r^ | [44] |
| pME4510lacI^q^P_tac_ | pME4510 carrying *lacI*^q^ and the P*_tac_* promoter | This study |
| pP_tac_PrrF2 | pME4510 harbouring PrrF2 under transcriptional control of P*_tac_* | This study |
| pME4510hfq_Flag_ | pME4510 carrying PAO1 *hfq* fused to a Flag-tag encoding sequence under control of its authentic promoter | [3] |
| pME4510hfq_Y25D_ | pME4510hfq_Flag_ encoding the PAO1 Hfq_Y25DFlag_ protein | [3] |
| pME4510hfq_K56A_ | pME4510hfq_Flag_ encoding the PAO1 Hfq_K56AFlag_ protein | [3] |
| pUC19 | *colE1 ori*; Ap^r^ | [45] |
| pHfq_Pae_ | pUC19 derivative harbouring PAO1 *hfq* under transcriptional control of P*_lac_* and translational control of the T7 gene *10* RBS | [3] |
| pHfq_PaeFlag_ | pUC19 derivative harbouring PAO1 *hfq_Flag_* under transcriptional control of P*_lac_* and translational control of the T7 gene *10* RBS | This study |
| pHfq_PaeY25DFlag_ | pHfq_PaeFlag_ encoding the PAO1 Hfq_Y25DFlag_ mutant protein | This study |
| pHfq_PaeK56A_ | pHfq_Pae_ encoding the PAO1 Hfq_K56A_ mutant protein | [3] |
| pET22b | T7 expression vector, encodes His-tag; Ap^r^ | Novagen |
| pMMB67HE | IncQ expression vector carrying an inducible P*_tac_* promoter, Ab/Cb^r^ | [46] |
| pMMBcrcZ | pMMB67HE harboring *crcZ* under transcriptional control of P*_tac_* | [3] |
| pME3087 | Suicide vector, ColE1 replicon, Mob; Tc^r^ | [47] |
| pME3087Δ*hfq* | pME3087 with a 246-bp deletion of *hfq* (nt +1 to nt +246 with regard to the A (+1) of the start codon) | This study |
