# Supplementary material for: The Pseudomonas aeruginosa CrcZ RNA interferes with Hfq-mediated riboregulation
Source: PLoS One. 2017 Jul 7;12(7):e0180887. doi: 10.1371/journal.pone.0180887 (PMC5501646; doi:10.1371/journal.pone.0180887)
Supplement: S1 Text — (DOCX) [file pone.0180887.s002.docx]

**Supporting Materials and methods**

**Construction of plasmids**

**Plasmid pTLantR**

Plasmid pTLantR bearing the *antR::lacZ* translational reporter gene was constructed as follows. A 342-bp fragment (nt -311 to nt +31 with regard to the A (+1) of the start codon) including the *antR* promoter was amplified by PCR using the oligonucleotides B48 (5’-TTT TGA ATT CCT CAC CCT TGT GCG TTG TCG-3’) and C48 (5’-TTT TCT GCA GCA CGA TCG GCG ACG GGA TG-3’) and chromosomal DNA of PAO1 as template. The PCR fragment was cleaved with *Eco*RI and *Pst*I, and then ligated into the corresponding sites of plasmid pME6014, abutting the 7^th^ codon of *hfq* to the 8^th^ codon of *lacZ*.

**Plasmid pTCantR2**

To construct a transcriptional gene fusion between *antR* and *lacZ*, a 258-bp fragment containing the *antR* promoter region (nt -311 to nt -53 with regard to the A (+1) of the start codon) was amplified by PCR using the oligonucleotides B48 (5’-TTT TGA ATT CCT CAC CCT TGT GCG TTG TCG-3’) and K74 (5’-TTT TTT CTG CAG TTC AGC AAA ATG CAA GGC-3’) and chromosomal DNA of PAO1 as template. The PCR fragment was cleaved with *Eco*RI and *Pst*I and then ligated into the corresponding sites of plasmid pME6016.

**Plasmid pME4510lacI^q^P_tac_**

To introduce an inducible P_tac_ promoter into the multiple cloning site of pME4510 a 1455-bp fragment containing the *lac*I^q^ gene was amplified by PCR using the oligonucleotides L85 (5’-GAT ATC GAA TTC GAA CGC CAG CAA GAC-3’) and Q107 (5’-GAT ATC CCC GGG **ATT ATA** TTG TTA TCC GCT CAC AA**T GTC** **AA**T TGT TAT CCG CTC ACA ATT CAG AAT ATT TGC CAG AAC CG-3’) and plasmid pMMB67HE as template. Oligonucleotide Q107 contained the sequence of the P_tac_ promoter (in bold). The PCR fragment was cleaved with *Eco*RI and *Sma*I and then ligated into the corresponding sites of plasmid pME4510.

**Plasmid pP_tac_PrrF2**

In plasmid pP_tac_PrrF2 the *prrF*2 gene was placed under the transcriptional control of the P*_tac_* promoter. A 130-bp fragment (coordinates 5284206 to 5284319 in the PAO1 genome [38]) was amplified by PCR using the oligonucleotides B127 (5´-CCC GGG ACT GGT CGC GAG GCC AG-3´) and H120 (5´-TTT TTT TCT GCA GGC TAC GTT TTC GCG GGC-3´) and chromosomal DNA of PAO1 as template. The PCR fragment was cleaved with *Pst*I and *Sma*I and then ligated into the corresponding sites of plasmid pME4510lacI^q^P_tac_.

**Plasmids pHfq_PaeFlag_ and pHfq_PaeY25DFlag_**

To construct pHfq_PaeY25DFlag_, a 246-bp fragment of *hfq* (nt +1 to nt +246 with regard to the A (+1) of the start codon) was amplified by PCR using the oligonucleotides P47 (5’-GC**T CTA GA**A ATA TAA TAG TTT AAC TTT *AAG AAG GAG ATA TAC ATA* TGT CAA AAG GGC ATT CGC TAC AAG ACC-3’) and F80 (5’-AA**G AAT TC**t cac ttg tcg tca tcg tct ttg tag tcA GCG TTG CCC GGC TCG) and chromosomal DNA of PAO1 as template. Oligonucleotide P47 contained a sequence derived from plasmid pET22b including the *Xba*I site (in bold) and the ribosome binding site of phage T7 gene *10* (shown in italics). Oligonucleotide F80 contained an *Eco*RI restriction site and a Flag-tag encoding sequence (depicted in lower case letters). The resulting PCR product was cleaved with *Xba*I and *Eco*RI and then ligated into the corresponding sites of plasmid pUC19. The resulting plasmid pHfq_PaeFlag_ harbours *hfq* under control of P_lac_ (derived from pUC19) and translation is directed by the ribosome binding site of T7 gene *10*. Then, the Y25D mutation was introduced into the *hfq*_Flag_ coding region by site directed mutagenesis using the QuickChange site-directed mutagenesis protocol (Agilent Technologies). The plasmid pHfq_PaeFlag_ was used together with the mutagenic oligonucleotides N81 (5’-GGT TTC CAT CGA TCT GGT CAA C-3’) and O81 (5’-GTT GAC CAG ATC GAT GGA AAC C-3’). The entire plasmid was amplified with Pfu DNA polymerase (Thermo Scientific). The parental plasmid template was digested with *Dpn*I and the mutated nicked circular strand was transformed into *E. coli* XL1-Blue, generating pHfq_PaeY25DFlag_.

**Construction of Plasmid pME3087Δ*hfq.***

To construct an in frame deletion of *hfq* we used the following procedure. Two PCR products flanking the *hfq* gene were obtained from PAO1 chromosomal DNA with primer pairs H71/J71 (H71: 5’-TTT TTT T**GG ATC C**GA TCC CGA GGT GCG CGC; J71: 5’-GTG CCG CAC TCC TTT AAG G-3’) and K71/I71 (K71: 5’-CCT TAA AGG AGT GCG GCA CTG ACG GGA GTC CGC TTT G-3’; I71: 5’-TTT TTT T**GA ATT C**GC GGC AGG TGG CGG-3’), respectively. The combined 717-bp upstream and 799-bp downstream fragments were used as a template for a second overlapping PCR with primers H71 and I71, which was possible because the primers J71 and K71 carry a complementary sequence. The resulting fragment with a 246-bp deletion, which spans the entire *hfq* coding region except for the stop codon, was digested with *Bam*HI and *Eco*RI and ligated into the corresponding sites of the suicide vector pME3087.

All DNA manipulations were verified by DNA sequencing.

**Generating of a markerless PAO1*Δhfq* strain**

The strain PAO1Δ*hfq* was constructed by homologous recombination. Briefly, plasmid pME3087Δ*hfq* was mobilized into strain PAO1 with the aid of *E. coli* strain HB101(pRK2013) [39], and then chromosomally integrated through selection for tetracycline resistance. Excision of the vector by a second crossover event was achieved by enrichment for tetracycline-sensitive cells [40].

**Determination of PrrF stability**

The stability of PrrF RNA was determined upon addition of rifampicin (100 µg/ml final concentration) to PAO1 and PAO1Δ*hfq* grown to an OD_600_ of 1.5 in BSM medium supplemented with 40 mM succinate. Samples were withdrawn at several times thereafter (see S2 Fig). Total RNA was extracted by using the hot phenol method [35]. 3 µg of total RNA was used for determining the levels of PrrF and 5S rRNA (loading control) by Northern-blot analysis as described in Materials and Methods.

**FRET assays**

Experimental details on the FRET assays have been described [16,41]. Briefly, two complementary, fluorophore-tagged RNA 21mers (Cy5-5′-AUG UGG AAA AUC UCU AGC AGU-3′ (Cy5-21R^+^) and Cy3-5′-ACU GCU AGA GAU UUU CCA CAU-3′ (Cy3-21R^−^)) were used. The tagged RNA oligonucleotides were purchased from VBC-Biotech (Vienna, Austria). Using a Tecan GENios Pro microplate reader, the first oligoribonucleotide was injected into the wells with or without Hfq protein (100 nM final Hfq-hexamer concentration), and the measurement was started with the injection of the second oligoribonucleotide. The reaction was performed in annealing buffer (50 mM Tris-HCl pH 7.5, 3 mM MgCl_2_ and 1 mM DTT) at 37°C. The final concentration of the RNAs was 5 nM in a volume of 40 µl. The reaction was allowed to proceed for 180 seconds, and with Cy3 excited, donor and acceptor dye fluorescence emissions were measured once every second. The time-resolved ratio of the fluorescence emissions (FRET index F_Cy5_/F_Cy3_) was normalized to 1 at t_180s_ and least-square fitted with Prism 4.03 (GraphPad Software Inc., San Diego, CA, USA) with the second-order reaction equation for equimolar initial reactant concentrations: *y* = *A*[1-(*k_obs_* *t*+1)^-1^], where *y* = fraction annealed, *k_obs_* = observed annealing reaction constant, *A* = maximum reaction amplitude. The observed reaction constants *k_obs_* were calculated as the average of three individually fitted reactions.

**Supplementary References**

1. Winsor GL, Griffiths EJ, Lo R, Dhillon BK, Shay JA, Brinkman FS. Enhanced annotations and features for comparing thousands of *Pseudomonas* genomes in the *Pseudomonas* genome database. Nucleic Acids Res. 2016; 44: D646-53.
2. Figurski DH, Helinski DR. Replication of an origin-containing derivative of plasmid RK2 dependent on a plasmid function provided in trans. Proc Natl Acad Sci U S A. 1979; 76: 1648-1652.
3. Ye RW, Haas D, Ka JO, Krishnapillai V, Zimmermann A, Baird C, et al. Anaerobic activation of the entire denitrification pathway in *Pseudomonas aeruginosa* requires Anr, an analog of Fnr. J Bacteriol. 1995; 177: 3606-3609.
4. Rajkowitsch L, Schroeder R. Coupling RNA annealing and strand displacement: a FRET-based microplate reader assay for RNA chaperone activity. Biotechniques. 2007; 43: 304-310.
5. Holloway BW, Krishnapillai V, Morgan AF. Chromosomal genetics of *Pseudomonas*. Microbiol Rev. 1979; 43: 73-102.
6. Schnider-Keel U, Seematter A, Maurhofer M, Blumer C, Duffy B, Gigot-Bonnefoy C, et al. Autoinduction of 2,4-diacetylphloroglucinol biosynthesis in the biocontrol agent *Pseudomonas fluorescens* CHA0 and repression by the bacterial metabolites salicylate and pyoluteorin. J Bacteriol. 2000; 182: 1215-1225.
7. Rist M, Kertesz MA. Construction of improved plasmid vectors for promoter characterization in *Pseudomonas aeruginosa* and other gram-negative bacteria. FEMS Microbiol Lett. 1998; 169: 179-183.
8. Yanisch-Perron C, Vieira J, Messing J. Improved M13 phage cloning vectors and host strains: nucleotide sequences of the M13mp18 and pUC19 vectors. Gene. 1985; 33: 103-119.
9. Fürste JP, Pansegrau W, Frank R, Blöcker H, Scholz P, Bagdasarian M, et al. Molecular cloning of the plasmid RP4 primase region in a multi-host-range *tac*P expression vector. Gene. 1986; 48: 119-131.
10. Voisard C, Bull CT, Keel C, Laville J, Maurhofer M, Schnider U, et al. Biocontrol of root diseases by *Pseudomonas fluorescens* CHA0: current concepts and experimental approaches. In: O’Gara F, Dowling D, Boesten B, editors. Molecular Ecology of Rhizosphere Microorganisms. Weinheim (Germany): VCH Publishers; 1994. p. 67-89.
