# Supplementary figures and images for: The Pseudomonas aeruginosa CrcZ RNA interferes with Hfq-mediated riboregulation
Source: PLoS One. 2017 Jul 7;12(7):e0180887. doi: 10.1371/journal.pone.0180887 (PMC5501646; doi:10.1371/journal.pone.0180887)

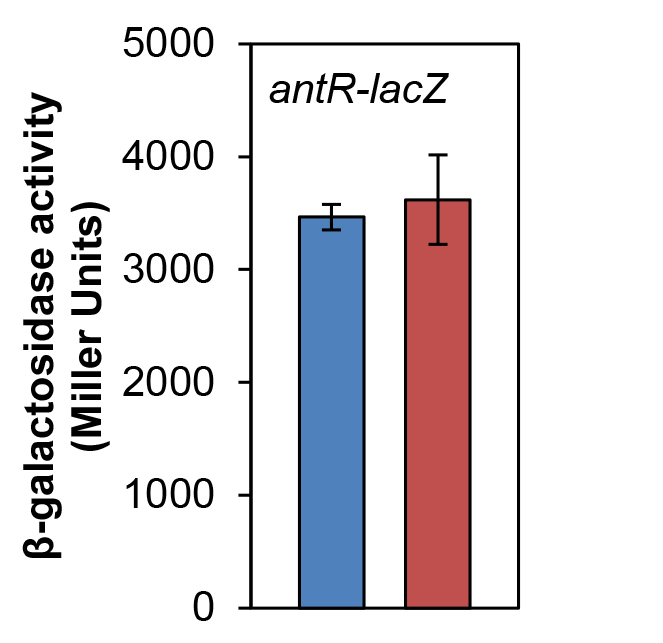

Supplement: S1 Fig — The strains PAO1(pTCantR2) and PAO1Δhfq(pTCantR2) were grown in BSM medium supplemented with 40 mM succinate and 0.2 mM anthranilate. When compared with the wild-type strain (blue bar) no significant difference in the β-galactosidase activity conferred by the transcriptional antR-lacZ fusion gene was observed in the PAO1Δhfq strain (red bar). (TIF) [file pone.0180887.s003.tif]

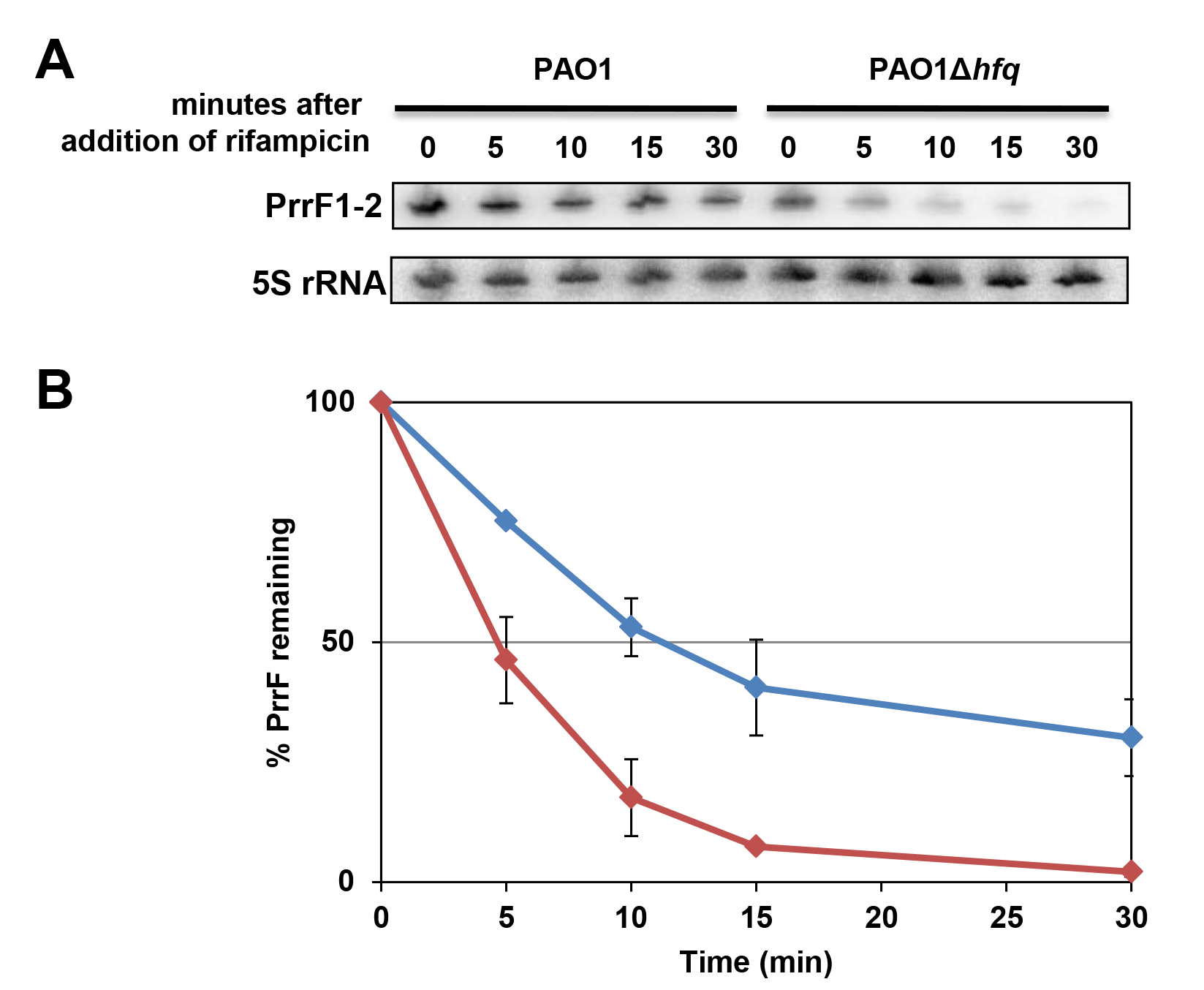

Supplement: S2 Fig — PAO1 and PAO1Δhfq were grown in BSM medium supplemented with 40 mM succinate. At an OD600 of 1.5, rifampicin was added to a final concentration of 100 μg/ml and samples were withdrawn for total RNA extraction at the times indicated. (A) The levels of PrrF1-2 and 5S rRNA (loading control) were determined by Northern-blot analyses with oligonucleotides specific for either RNA as described in Materials and methods. The result from one representative experiment is shown. (B) Graphical representation of the results. The concentrations of PrrF1-2 RNA in PAO1 (blue diamonds) and PAO1Δhfq (red diamonds), respectively, were normalized to that of 5S rRNA at different times after addition of rifampicin. The results are derived from two independent experiments. Error bars represent standard deviations. The half-life of PrrF1-2 RNA was determined with 12 ± 3 min in PAO1 and 5 ± 1 min in PAO1Δhfq. (TIF) [file pone.0180887.s004.tif]

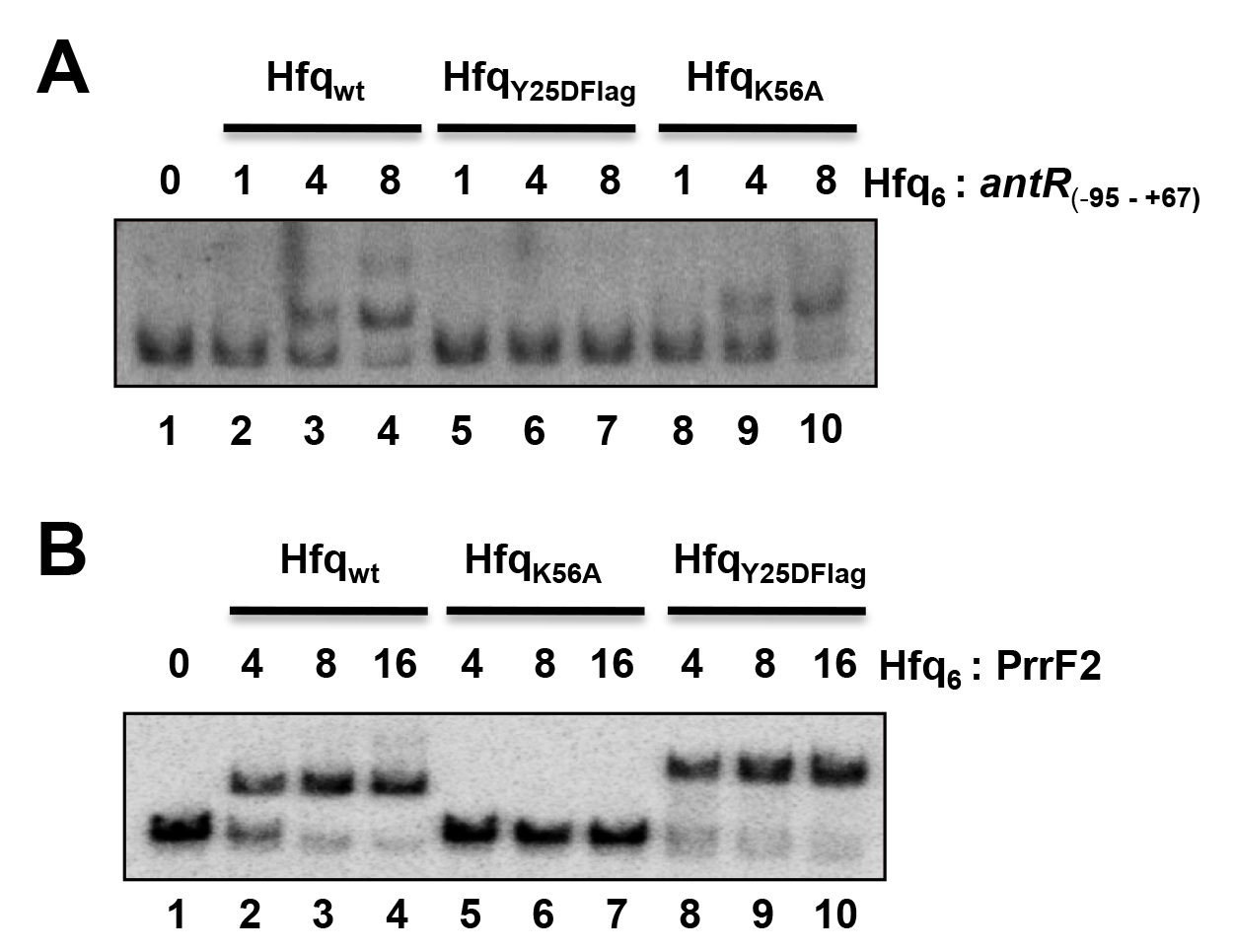

Supplement: S3 Fig — (A) EMSA with 10 nM radioactively labeled antR(-95–+67) mRNA in the absence (lane 1) and in the presence of 1-fold (lanes 2, 5 and 8), 4-fold (lanes 3, 6 and 9) and 8-fold (lanes 4, 7 and 10) molar excess of HfqPae (Hfqwt), HfqPaeY25DFlag and HfqPaeK56A, respectively. (B) EMSA with 10 nM radioactively labeled PrrF2 sRNA in the absence (lane 1) and in the presence of 4-fold (lanes 2, 5 and 8), 8-fold (lanes 3, 6 and 9) and 16-fold (lanes 4, 7 and 10) molar excess of HfqPae (Hfqwt), HfqPaeY25DFlag and HfqPaeK56A, respectively. (TIF) [file pone.0180887.s005.tif]

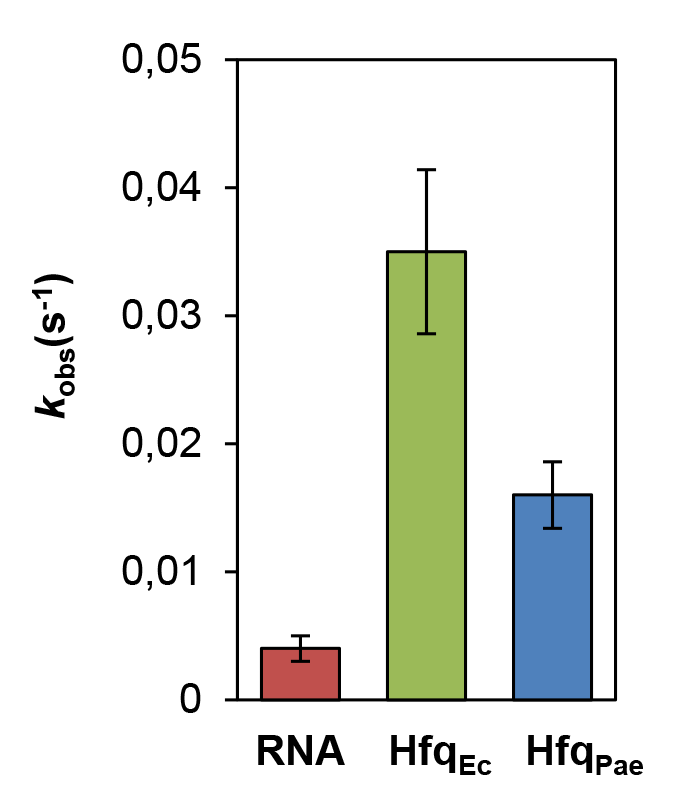

Supplement: S4 Fig — RNA annealing activities of HfqPae and HfqEc. 5 nM single stranded, complementary 21-nt-long oligonucleotides with fluorophores at their 5’-end were annealed at 37°C in the absence (red bar) or presence of 100 nM HfqEc (green bar) and HfqPae (blue bar) protein, respectively. Relative fluorescence resonance energy transfer (FRET) was calculated as ratio of acceptor to donor fluorescence (FCy5/FCy3) as described in S1 Text. The time-resolved curves were least-square fitted with the second-order reaction equation for equimolar initial reactant concentrations: y = A[1-(kobs t+1)-1]; y = fraction annealed, kobs = observed annealing reaction constant, A = maximum reaction amplitude. The reaction rate kobs was calculated from the average of three independent experiments. (TIF) [file pone.0180887.s006.tif]

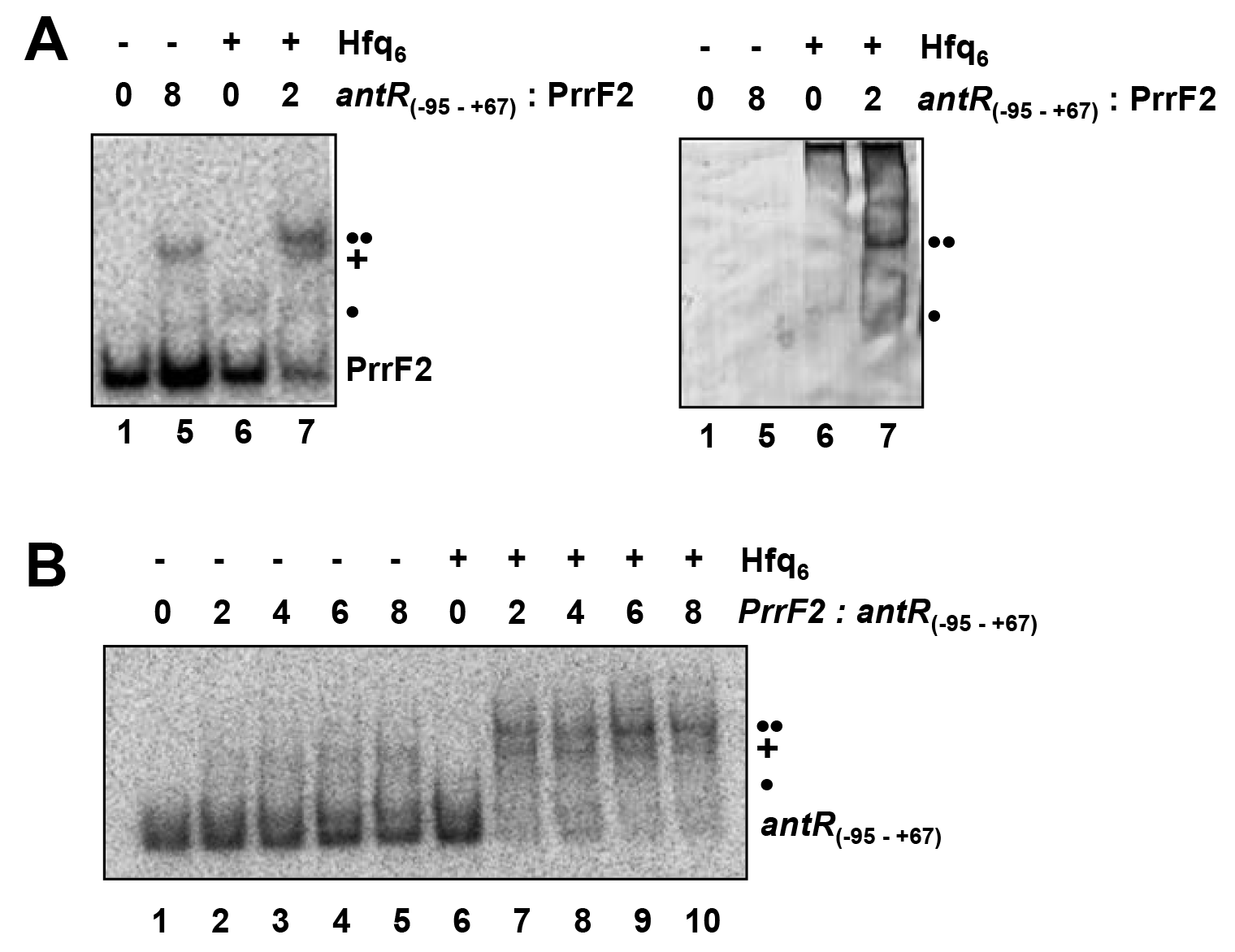

Supplement: S5 Fig — (A) Left panel: The samples corresponding to those shown in Fig 1C, lanes 1, 5, 6 and 7 were resolved on a separate 4% native polyacrylamide gel. Single and double circles denote the PrrF2·Hfq and PrrF2·Hfq·antR(-95–+67) complexes, respectively. The plus symbol denotes the PrrF2·antR(-95–+67) complex. Right panel: Western-blot of the gel shown at the left probed with anti-Hfq antibodies. The presence of Hfq in the PrrF2·Hfq and PrrF2·Hfq·antR(-95–+67) complexes is indicated by single and double circles, respectively. (B) Hfq accelerates PrrF-antR duplex formation. 10 nM radioactively labeled antR(-95–+67) RNA was incubated alone (lane 1), with HfqPae (lane 6) or with increasing amounts of PrrF2 (2, 4, 6 and 8-fold molar excess) in the absence (lanes 2–5) or presence (lanes 6–10) of HfqPae (the molar ratio of HfqPae-hexamer to antR(-95–+67) RNA to was 2:1), and the resulting complexes were analyzed on a 4% native polyacrylamide gel. Single and double circles denote the antR(-95–+67)·Hfq and PrrF2 Hfq·antR(-95–+67) complexes, respectively. The plus symbol denotes the PrrF2·antR(-95–+67) complex. (TIF) [file pone.0180887.s007.tif]
